# Supplementary material for: Screening and Identification of Goat-Milk-Derived Lactic Acid Bacteria with Bacteriocin-like Activity and Probiotic Potentials
Source: Microorganisms. 2023 Mar 27;11(4):849. doi: 10.3390/microorganisms11040849 (PMC10143788; doi:10.3390/microorganisms11040849)
Supplement: Supplementary file 1 [file microorganisms-11-00849-s001.zip › microorganisms-2290433-supplementary.pdf]

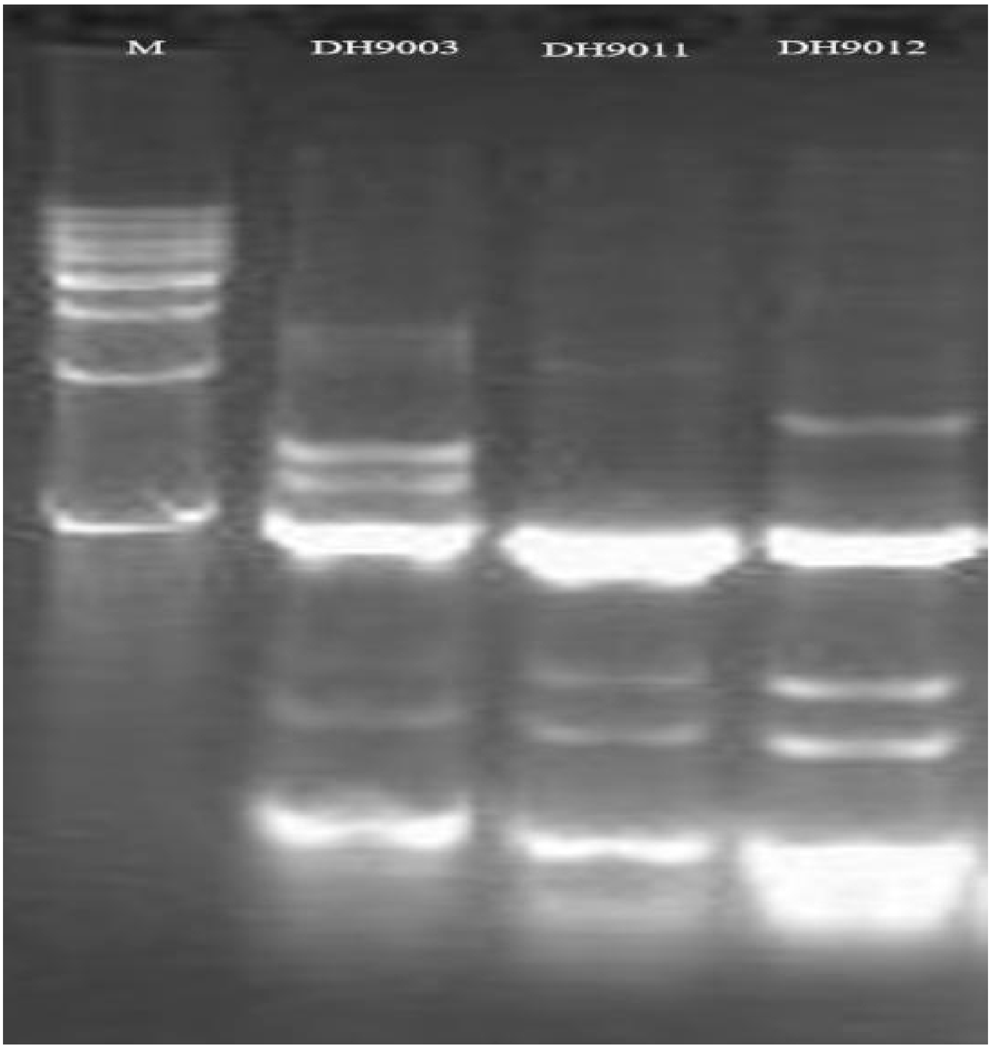

Figure S1. Repetitive-PCR products of three bacteriocin producers from lactic acid bacteria via agarose gel electrophoresis.
